# Shedding Light on Dark Chemical Matter: The Discovery of a SARS-CoV-2 M^pro^ Main Protease Inhibitor through Intensive Virtual Screening and In Vitro Evaluation

**DOI:** 10.3390/ijms25116119

**Published:** 2024-06-01

**Authors:** Maria Nuria Peralta-Moreno, Yago Mena, David Ortega-Alarcon, Ana Jimenez-Alesanco, Sonia Vega, Olga Abian, Adrian Velazquez-Campoy, Timothy M. Thomson, Marta Pinto, José M. Granadino-Roldán, Maria Santos Tomas, Juan J. Perez, Jaime Rubio-Martinez

**Affiliations:** 1 Department of Materials Science and Physical Chemistry, Institut de Recerca en Quimica Teòrica i Computacional (IQTCUB), University of Barcelona (UB), 08028 Barcelona, Spain; peralta.mnuria@ub.edu (M.N.P.-M.); yago.mena@gmail.com (Y.M.); 2 Institute of Biocomputation and Physics of Complex Systems (BIFI), Joint Unit GBsC-CSIC-BIFI, Universidad de Zaragoza, 50018 Zaragoza, Spain; dortega@bifi.es (D.O.-A.); ajimenez@bifi.es (A.J.-A.); svega@bifi.es (S.V.); oabifra@unizar.es (O.A.); adrianvc@unizar.es (A.V.-C.); 3 Departamento de Bioquímica y Biología Molecular y Celular, Universidad de Zaragoza, 50009 Zaragoza, Spain; 4 Instituto de Investigación Sanitaria de Aragón (IIS Aragon), 50009 Zaragoza, Spain; 5 Centro de Investigación Biomédica en Red en el Área Temática de Enfermedades Hepáticas Digestivas (CIBERehd), 28029 Madrid, Spain; titbmc@ibmb.csic.es; 6 Institute of Molecular Biology of Barcelona (IBMB-CSIC), 08028 Barcelona, Spain; 7 Instituto de investigaciones de la Altura, Universidad Peruana Cayetano Heredia, Av. Honorio Delgado 430, Lima 15102, Peru; 8 AbbVie Deutschland GmbH & Co. KG, Computational Drug Discovery, Knollstrasse, 67061 Ludwigshafen, Germany; marta.pinto@abbvie.com; 9 Departamento de Química Física y Analítica, Facultad de Ciencias Experimentales, Universidad de Jaén, Campus “Las Lagunillas” s/n, 23071 Jaén, Spain; jmroldan@ujaen.es; 10 Department of Architecture Technology, Universitat Politecnica de Catalunya (UPC), Av. Diagonal 649, 08028 Barcelona, Spain; maria.santos.tomas@upc.edu; 11 Department of Chemical Engineering, Universitat Politecnica de Catalunya (UPC), Barcelona Tech. Av. Diagonal, 647, 08028 Barcelona, Spain; juan.jesus.perez@upc.edu

**Keywords:** SARS-CoV-2 main protease, dark chemical matter, docking, molecular dynamics, MMPB/GBSA approach, drug design, virtual screening

## Abstract

The development of specific antiviral therapies targeting SARS-CoV-2 remains fundamental because of the continued high incidence of COVID-19 and limited accessibility to antivirals in some countries. In this context, dark chemical matter (DCM), a set of drug-like compounds with outstanding selectivity profiles that have never shown bioactivity despite being extensively assayed, appears to be an excellent starting point for drug development. Accordingly, in this study, we performed a high-throughput screening to identify inhibitors of the SARS-CoV-2 main protease (M^pro^) using DCM compounds as ligands. Multiple receptors and two different docking scoring functions were employed to identify the best molecular docking poses. The selected structures were subjected to extensive conventional and Gaussian accelerated molecular dynamics. From the results, four compounds with the best molecular behavior and binding energy were selected for experimental testing, one of which presented inhibitory activity with a *K_i_* value of 48 ± 5 μM. Through virtual screening, we identified a significant starting point for drug development, shedding new light on DCM compounds.

## 1. Introduction

Sparked by a previously unidentified coronavirus known as SARS-CoV-2, an outbreak of respiratory illness referred to as COVID-19 developed in December 2019 [1]. With the first cases having originated in Wuhan, Hubei Province, China, the rapid spread of infections around the world triggered a global health emergency that urged the World Health Organization to declare it as an infectious disease pandemic of international concern on 11 March 2020 [2]. Social measures and widespread testing, combined with the rapid development of an efficacious vaccine, helped to lift the acute phase of the pandemic on 5 May 2023 [3]. Despite this accomplishment, COVID-19 continues to represent a global health burden and is still a cause of death in many parts of the world [4].

Despite the positive impact of vaccination, the rapid evolution of the virus, waning immunity, and the high cost of vaccination makes the identification of effective antivirals a high priority. Several antiviral agents, approved under emergency authorization, are presently available. Current standard-of-care antivirals against COVID-19 include the SARS-CoV-2 main protease (M^pro^) inhibitor, Nirmatrelvir [5] (co-packaged with the CYP3A inhibitor Ritonavir and sold as Paxlovid^®^ [6]), and the RNA-dependent RNA polymerase inhibitor, Remdesivir [7,8], shown to be efficacious in viral clearance and in preventing severe disease when administered in a timely manner. In addition, Molnupiravir [9], an oral nucleotide analog with broad-spectrum antiviral activity, is no longer recommended in most countries due to its ineffectiveness and induction of counterproductive mutagenesis [10]. Other repurposed antivirals, such as Lopinavir/Ritonavir, also proved to be ineffective in treating the illness [11]. The need to rapidly discover specific antiviral therapies against SARS-CoV-2 makes drug repurposing a preferred strategy in many COVID-19 drug discovery initiatives [12,13,14,15,16,17]. Moreover, computational chemistry studies are a cost-effective strategy, as has been shown in many recently published studies. A few of them have focused on the virtual screening of FDA-approved compounds with the successful identification of drugs presenting inhibitory activity regarding coronavirus in vitro [18,19]. Natural products have also been considered as promising starting points in the discovery process of antiviral agents, including those identified by virtual screening and subsequent experimental in vitro validation for their inhibitory profile in regard to the SARS-CoV-2 M^pro^ [20,21,22,23,24].

From the early stages of drug design to subsequent hit-to-lead optimization studies, computational methodologies constitute an important tool in the development of new treatments [25]. One of these methodologies is virtual screening, where the structure of thousands of compounds is scrutinized to fulfill a specific set of stereochemical features. A key ingredient in efficiently performing virtual screening studies concerns the availability of structural databases alongside easy access to the compounds identified for experimental validation. Currently, there is a vast array of compound structure databases for drug design, such as the European Molecular Biology Laboratory ChEMBL database [26], which consists of a manually curated chemical database of bioactive molecules with drug-like properties, or the popular ZINC database [27,28].

In this work, we used the Dark Chemical Matter (DCM) database to conduct virtual screening studies [29]. This is a particularly interesting database of drug-like compounds that, despite presenting excellent selectivity profiles, have never shown biological activity before. Despite being extensively explored with no favorable experimental results so far, its use remains a promising starting point for drug development [30,31,32].

Accordingly, in the present study, we report the results of a virtual screening study of the DCM database aimed at identifying prospective inhibitors of the SARS-CoV-2 M^pro^ main protease. For this purpose, we employed a robust protocol previously developed in our group that shows reasonable success rates [18,20,21]. Specifically, two distinct and independent protocols of ensemble molecular docking were applied to identify inhibitors from the DCM database targeting different representative structures of the enzyme. After the identification and selection of the best candidates, extensive molecular dynamics calculations were conducted to evaluate the free binding energy of a selected set of complexes. Finally, compounds with the best binding affinities and energy profiles were selected and procured for in vitro testing, with one of the four compounds assessed showing a promising M^pro^ inhibitory profile.

## 2. Results

### 2.1. Virtual Screening Targeting the SARS-CoV-2 M^pro^ Main Protease

To undertake the ensemble molecular docking of the diverse compounds in the Dark Chemical Matter database, seven representatives of the SARS-CoV-2 M^pro^ monomeric structure were used. The ensemble molecular docking was performed using the QVina2 software QuickVina 2 version [33]. To ensure a varied selection of compounds with different ligand sizes, an additional size-independent docking protocol was implemented to complement the information extracted from the original approach. Thus, two independent strategies were conducted. In the first (hereafter, Dock1), we applied the default scoring function of QVina2 to rank ligand-M^pro^ complexes, while for the second (hereafter, Dock2) we introduced a correction to obtain a size-independent scoring function. Specifically, in Dock2, the QVina2 default scoring function was adjusted by dividing it by a ${Num\_NoH}^{0.3}$ factor, where *Num_NoH* corresponds to the number of non-hydrogen atoms of the ligand [34].

For each representative structure, ligand-M^pro^ complexes presenting a scoring function value lower than or equal to an established threshold were rank ordered and saved for further analysis. Minimum and maximum values are reported in Table S1. A −8.1 kcal/mol threshold was established for Dock1 after an in-depth analysis of the resulting energy distribution (Table S2). This number was selected to include chemical diversity and to keep the computational cost reasonable. A different threshold was used in Dock2 for each representative (Table S2) in order to keep the number of selected poses around 200. In this case, the range of energies generated in the docking process was small compared to that obtained using the non-modified scoring function, making a practical selection with a single threshold difficult. As a result, a total of 3625 and 1012 complexes were selected for Dock1 and Dock2, respectively. However, it is important to note that the selected complexes may include more than one pose per compound, and some of them may appear on the list of different representatives (Table S3).

After the selection of the ligand–receptor complexes for both docking processes, a minimization protocol was implemented. At this point, we introduced explicit solvation and allowed conformational accommodation for both ligand and receptor. Since molecular docking is a technique that enables the generation of diverse poses of the compounds to target the receptor under study, some may present steric clashes, bad contacts, or bad atom orientations in the three-dimensional conformational space. Hence, a minimization process is an essential step in obtaining more relaxed complex structures to proceed further with the study.

The binding free energies of the minimized structures were obtained by the application of the MMPBSA and MMGBSA end-point methodologies [35], respectively, denoted as Δ*G*_binding_(PB) and Δ*G*_binding_(GB). At the end of both processes, an independent rank ordered list was obtained for each representative structure of M^pro^. As a result, a total of 28 lists were obtained, 1 for each of the 7 representative structures using 2 different approaches for energy calculation (PB/GB) for each of the 2 independent docking processes.

At this stage, compound selection for subsequent molecular dynamic studies becomes essential. Rather than focusing only on compounds exhibiting the best binding affinities, our approach also prioritizes those ligands able to bind to different conformations of the target protein. Consequently, we employed a consensus approach that also considers the assumption that the larger the number of receptor conformations a ligand binds, the higher its chances of success. According to this criterion, 106 compounds were selected for Dock1 and 90 for Dock2. In the first case, we included those compounds that exhibit binding to six, five, four, and three representatives of the target. No compound was found capable of binding to all receptors. In the second case, compounds that exhibit binding to four, three, and two representatives of the target were included. No compound was found capable of binding to seven, six or five receptors. For each compound, the complex structure with the lowest binding energy was selected for further studies.

Next, the selected compounds were subjected to a preparation step. Accordingly, a 0 to 300 K heating process, followed by a density equilibration step, was carried out for each of the selected complexes. Thereafter, a production run of 100 ns of conventional Molecular Dynamics (cMD) simulations was performed.

To analyze the time evolution of the free binding energy, Δ*G*_binding,_ of all the initially selected compounds, the MMGBSA approach was employed. Following the previous procedures established by us [18,20,21], an iterative process, consisting of the extension of the simulation length of the previous molecular dynamics simulations, was performed for the analysis. During each iteration, complexes that exhibited a smooth Δ*G*_binding_ fluctuating behavior during the last 20 ns of the cMD simulation were kept to continue with the protocol. Thus, the cMD simulations of 72 and 48 complexes were extended to 200 ns for Dock1 and Dock2, respectively. Thereafter, 29 and 22 complexes were selected to extend their simulations to 500 ns for Dock1 and Dock2, respectively.

Next, to better study the stability of the selected complexes and to reduce the number of candidates, cMD was switched to Gaussian accelerated Molecular Dynamics (GaMD) (see the methodology Section 4.1.3 and Section 4.1.4 for more details). Consequently, GaMD simulations of 1 µs were undertaken for eight and five complexes selected from Dock1 and Dock2 protocols, respectively. Finally, four and three complexes were selected for Dock1 and Dock2, respectively, to extend their GaMD simulations up to 1.6 µs to check the stability of the previously observed free binding energy smooth behaviour (Table S4). The Δ*G*_binding_ (GB) time evolution plots for all seven selected complexes, obtained using the MMGBSA approach, are shown in Figures S1–S3.

Free binding energy profiles exhibiting minor fluctuations were identified as a robust indication of any compound to be selected for experimental testing. These fluctuations, linked to the ligand motion within the interaction hotspot, can be translated into complex stability. Therefore, good drug candidates are expected to present mild fluctuations, exhibiting constant and smooth binding energy profiles with small deviations, suggesting the stability of the binding pose during interaction. On the other hand, the compounds to be excluded from experimental assays will correspond to those exhibiting sharp changes in energy profiles, suggesting the possible pose misadaptation of the ligand to the binding site, despite presenting good binding energies. However, if these compounds eventually stabilize after the extension of the simulation, they can also be considered as candidates for experimental testing. As such, displaying smooth and stable energy profiles is required for selection, in addition to presenting good affinity values.

Following the analysis, the time evolution of all selected compounds binding energies was evaluated and found to be stable for all of them. More specifically, compounds DM2 and DM5–7 presented good convergence during the complete simulation, exhibiting small fluctuations not higher than approximately 20 kcal/mol of their average value (Figures S2 and S3). Similarly, compound DM1 also showed a strong converged profile, especially during the last 1.2 µs of extended GaMD simulation. Upon monitoring its time evolution plot (Figure S1), a sudden energy stabilization was observed before the first 400 ns of simulation, likely due to a conformational change caused by a pose adaptation of the ligand to increase its interactions and stability at the binding site. Despite some fluctuations in their energetic profiles, compounds DM3 and DM4 exhibited good affinities during the entire analysis process and were thus also selected for further experimental studies. Hence, after analyzing the binding stability and behavior of the time evolution plots, all 7 compounds were selected as prospective candidates for in vitro testing as a result of the extensive virtual screening process performed (Table S4).

### 2.2. In Vitro Activity Assay of Selected Candidate Compounds

From the selected potential candidates identified through the virtual screening described above, only four (two from each virtual screening protocol) were commercially available and consequently experimentally tested in an in vitro activity assay. 

**Figure 1:**
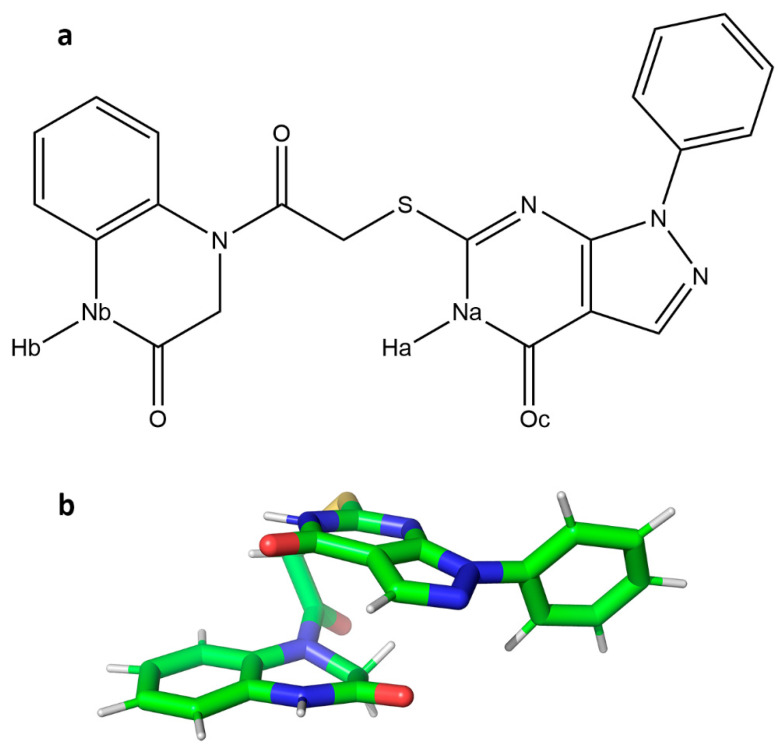
(**a**) Chemical structure of compound DM1 identified from the virtual screening process as a prospective hit targeting the SARS-CoV-2 M^pro^ main protease. (**b**) Three-dimensional representation of the compound in its bioactive conformation obtained from the extended Molecular Dynamics simulations. Carbon atoms are represented in green; nitrogen and oxygen atoms are respectively depicted in blue and red, and hydrogen atoms in white.

Among the tested candidates (Table S4), one compound, DM1 (Figure 1), showed specific inhibitory activity, exhibiting a substrate concentration-independent inhibition constant (*K_i_*) of 48 ± 5 µM (Figure S4). In contrast, the remaining three compounds tested did not yield detectable inhibitory activities at concentrations below 125 µM.

Based on the good energetic profiles shown by the selected but non-tested compounds, we also suggest experimentally assessing their potential inhibitory activity to target SARS-CoV-2 M^pro^.

### 2.3. Binding Analysis of the Active Compound

As also demonstrated in our previous studies [18,20,21], the docking protocol employed in the current study, exploring the DCM database, yielded at least one experimentally active compound among those selected as candidate inhibitors of the SARS-CoV-2 M^pro^ protease. Departing from the smooth energetic profile found for the active compound (Figure S1), a further binding analysis was performed.

**Figure 2:**
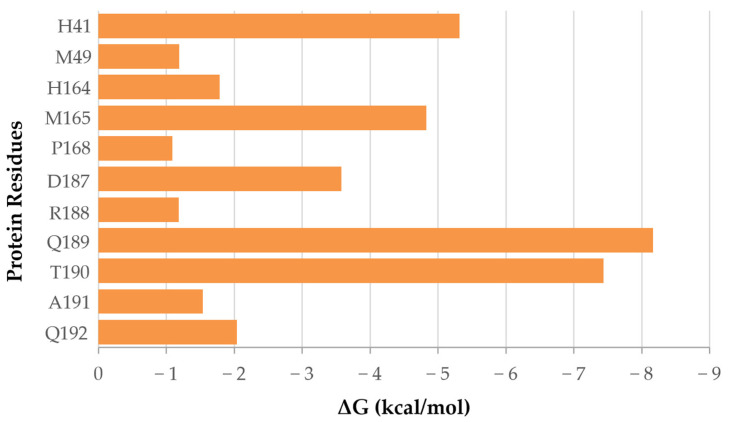
Residue decomposition of the binding free energy interaction extracted from the final 100 ns of the extended GaMD simulations performed for the DM1 ligand—SARS-CoV-2 M^pro^ protease complex, experimentally found to be active.

To assess for significant ligand–protein residue interactions in the complex, a free energy decomposition per residue analysis of the last 100 ns of the total 1.6 µs GaMD trajectory for the active compound was conducted (Figure 2 and Table S5). As such, we found major contributions of M^pro^ residues Q189 and T190 with energy contributions of −8.2 and −7.4 kcal/mol, respectively, involving hydrogen bond interactions with the ligand. Interactions between the catalytic dyad H41 and the ligand were significant, with a contribution of −5.3 kcal/mol, involving π-π interactions (Figure S5).

**Figure 3:**
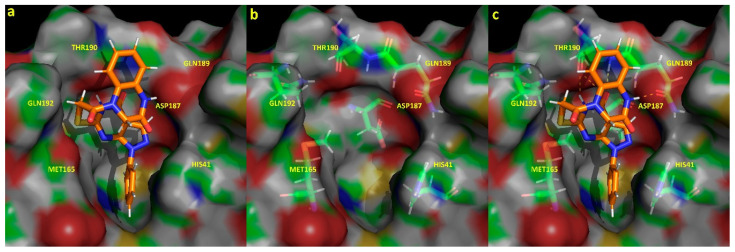
Binding site representation of the active dark chemical matter compound DM1 in complex with the SARS-CoV-2 M^pro^ protease, taken from the last frame of the extended GaMD simulation performed. Representation (**a**) shows the three-dimensional structure of the ligand interacting at the binding pocket, defined by residues H41, M165, D187, Q189, T190, and Q192. Representation (**b**) depicts the spatial distribution of the protein residues defining the pocket previously described. Complementary, hydrogen bonds established between the ligand and the protein are highlighted with yellow dashes (**c**). Carbon, nitrogen, oxygen, and hydrogen surface atoms are represented in green, blue, red, and white, respectively.

Furthermore, hydrogen bonds exhibiting a minimum of 90% occupancy during the last 100 ns of the extended 1.6 µs GaMD trajectory were evaluated (Table S6). From the results, acceptor–donor interactions between the Oc oxygen of the ligand (Figure 1) and residue T190, as well as interactions between the oxygen atoms of Q189 and T190 with ligand amino groups (Figure 3), were inferred to be major contributions defining the interactions of the active compound at the binding site. The three-dimensional structure of the active compound at the binding site, together with residues defining the pocket and subsequent interactions, are depicted in Figure 3. π-π interactions between the catalytic dyad residue H41 and the benzene group of DM1 (Figure S5) were found to contribute to the stability of the complex. Notably, the formation of intramolecular π-π interactions confer the ligand with its three-dimensional bioactive conformation.

## 3. Discussion

In response to the need for the development of specific antiviral therapies targeting SARS-CoV-2, numerous studies have focused on natural compounds, the repurposing of existing drugs or the massive screening of compounds. This work proposes an alternative virtual screening approach based on two independent ensemble docking strategies for the SARS-CoV-2 M^pro^ main protease and a set of drug-like compounds, stored in the Dark Chemical Matter (DCM) database, that have never shown bioactivity despite presenting outstanding selectivity profiles [29]. 

The selection of a docking score to be used to rank and identify the best compounds is the most important limitation of any virtual screening protocol. Consequently, two different docking scores were implemented as a major goal of this study in order to assess the goodness of the size-independency correction of the scoring function employed. Our approaches, both based on the default docking score provided by QVina2, differ in their consideration of a correction factor applied to only one of them so as to obtain a consensus based on size-independent binding affinities [34]. 

After the full completion of both independent protocols, a total of seven compounds were selected for in vitro testing as candidate inhibitors of M^pro^ activity, of which four corresponded to the original strategy and three to the corrected score. Of these compounds, four were subjected to experimental assays, one of them exhibiting significant inhibitory activity against M^pro^, thus shedding light onto the dark chemical matter library of compounds.

Compared with our group’s previous projects, in which the same main procedure was applied [18,20,21], similar results were obtained. The methodology, again, was sufficient to produce at least one experimentally active compound to inhibit the SARS-CoV-2 M^pro^ main protease. Thus, in this study, with one out of four tested compounds being active, we obtained a success rate of 25%.

Regarding interactions of the compound experimentally found to be active, compared to previously identified active compounds, such as (−) epigallocatechin gallate, amentoflavone, vitexin-2-*O*-rhamnoside, aloin, or rhoifolin [20], we must note the common binding site interactions with residues H41, M165, D187, and Q189 of SARS-CoV-2 M^pro^. Despite presenting differences in their specific free binding energy residue decomposition profiles, interactions with the catalytic dyad residue (H41) prove important in the binding and stability of the complex, especially concerning hydrogen bond formation, which, in general, can be translated into increased binding affinities and complex stability.

The catalytic mechanism of SARS-CoV-2 M^pro^ involves the alignment of residues H41 and C145 with the peptide substrate at the active substrate-binding pocket [36,37,38,39]. Therein, the drug candidate molecule will undertake a competitive binding process with the natural substrate of the protease. Then, only if favorable binding interactions exist, inhibitors of the Mpro will exhibit a higher affinity for the protein rather than for the substrate [36,40]. In this work, an in silico analysis of the active compound identified in vitro revealed interesting interactions with residue H41 but none with catalytic dyad C145. Jin, Z., Du, X., Xu, and Y. et al. identified up to four binding pockets inside the active site of SARS-CoV-2 M^pro^ [36]. Compared to our results, we can identify the significant interaction contributions of the active compound with H41, M49, D187, and R188, as well as with M165, P168, R188, T190, A191, and Q192, corresponding to residues defining binding pockets S2 and S4, as described in the literature by the authors.

In the end, our main virtual screening methodology suffices to facilitate an in vitro evaluation of candidate compounds, and is always able to at least identify one active compound [18,20,21]. Since the number of tested compounds from each independent protocol was small, it is very difficult to extract robust conclusions about the effect of the size-independency correction, or even to compare results. Also, it is important to mention that, for now, we have only introduced two different docking scores in the present work. Therefore, it will be interesting for prospective work to implement more varied functions and work with a broader consensus for results comparison. Furthermore, to truly assess the goodness of the approach, we encourage further studies to gather more experimental results for comparative analyses.

With the increase in computational resources and capabilities, it will also be interesting to combine different scoring functions, not only classical ones but also those supported by machine learning (ML) or artificial intelligence (AI), as well as to increase the number of replicates and the simulation length of the molecular dynamics to obtain more accurate and robust results.

## 4. Materials and Methods

### 4.1. Computational Studies

#### 4.1.1. SARS-CoV-2 M^pro^ Protease Representative Structures Selection

To introduce the conformational flexibility of the SARS-CoV-2 M^pro^ protease, seven selected M^pro^ representatives were used as receptors in the virtual screening process. We will provide a brief overview of the selection process, as previously described by our group [20]. The crystallographic structure of SARS-CoV-2 M^pro^ protease (PDB access code 6Y84), considering only the monomer, was prepared and placed in a cubic periodic box filled with four-point Optimal Point Charge (OPC) water molecules [41] with counterions to neutralize the charge of the unit cell using the Leap module of AMBER18 [42]. All calculations were carried out using the ff19SB force field [43]. Next, the structure was relaxed via a multistep minimization procedure to eliminate possible steric clashes [44]. Subsequently, independent duplicates of conventional Molecular Dynamics (cMD) and Gaussian accelerated Molecular Dynamics (GaMD) of 500 ns length were carried out to enhance the conformational space exploration of the system [45] within the NVT ensemble.

Finally, to select the most representative structures representing the greatest structural diversity of the binding site of the M^pro^ protease, a clustering process was performed separately for both cMD and GaMD calculations using the average linkage algorithm [46], implemented in the AMBER18 cpptraj module [42,47]. To conclude, seven representatives, three for the cMD and four for the GaMD, were selected to represent clusters with over 10% of the population.

#### 4.1.2. Virtual Screening

**Figure 4:**
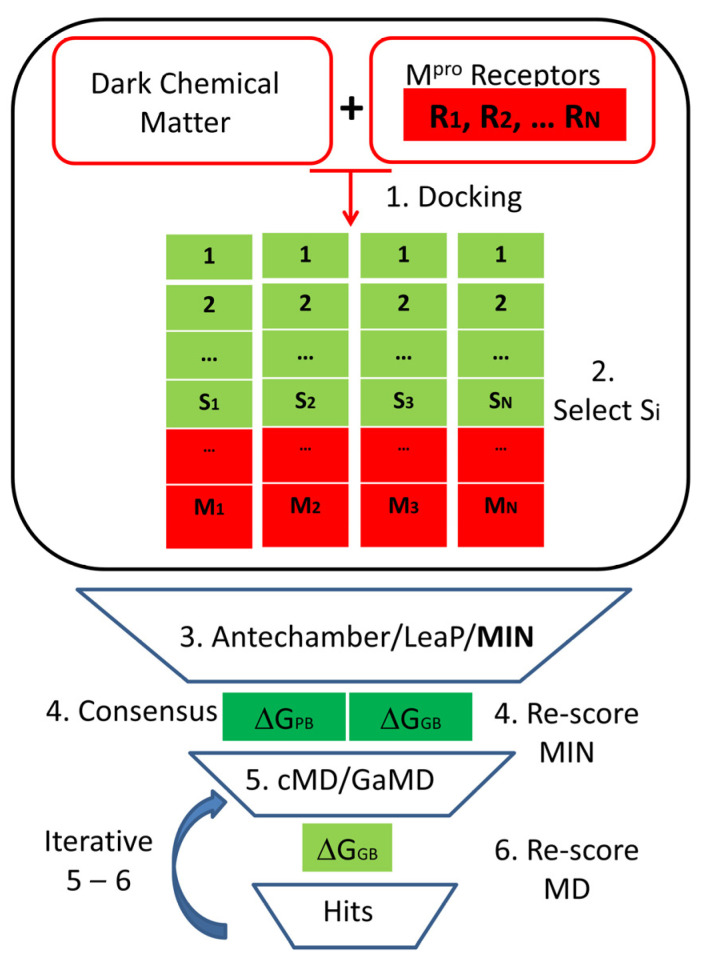
General flowchart of the multi-step Virtual Screening (VS) performed in the present work for the database of dark chemical matter (DCM) compounds.

For the seven SARS-CoV-2 M^pro^ representatives selected for the present work, two independent multi-step Virtual Screening (VS) processes were conducted, with and without using a scoring function correction for the ensemble molecular docking process. From now on, an overview of both protocols will be given (Figure 4).

Initially, in step 1, the QVina2 software [33] was employed to dock the molecules of the DCM database, composed of 76,962 compounds derived from the original database after preparation with OpenBabel (version 2.3.2) [48] and Antechamber module [49] included in AMBER18 software. In step 2, complexes presenting binding energy values higher than a predefined cut-off based on the energy function used to rank the docking results were selected for each M^pro^ representative structure. Subsequently, in step 3, the Antechamber and LeaP modules of the AMBER18 package [42] were used to parametrize the ligands with GAFF2 force field [50], add counterions, and solvate the complexes in a box of TIP3P water molecules [51]. To parametrize the protein, the ff14SB force field was employed [52]. Next, a multistep minimization procedure consisting of 5000 steps, each using the steepest descent method, was conducted to relax the whole system for each complex. First, all protein and ligand atoms were fixed by the application of harmonic positional restrictions of 5 kcal/mol·Å^−2^, only allowing relaxation for water molecules and ions. Then, as a subsequent step, only the main atoms of the protein were kept fixed with the same harmonic positional restrictions to allow the free movement of the ligand. Finally, in the last step of the minimization protocol, all atoms were allowed to move freely.

Then, in step 4, the free binding energy Δ*G*_binding_ of all minimized structures was computed using both the Molecular Mechanics Poisson–Boltzmann Surface Area (MMPBSA) and the Molecular Mechanics Generalized—Born Surface Area (MMGBSA) approaches [53,54]. These two values, using a consensus criterion, were used as a new scoring to rank and select the ligands that will be further studied by means of molecular dynamics simulations. Hence, in step 5, conventional Molecular Dynamics (cMD) or Gaussian accelerated Molecular Dynamics (GaMD) of increasing length were performed. After that, in step 6, a new ranking was again obtained after applying the MMGBSA approach to the full simulation length. Next, an iterative process involving steps 5 and 6 was performed. Consequently, at each iteration step, the extension of the simulation length, and the subsequent free binding energy recalculation, was only carried out for the previously selected compounds. In the end, after the selection of the best candidates, ligand–receptor interactions at the binding site were analyzed based on their free binding energies from the final extended simulations.

As previously mentioned, the difference between both independent protocols consists in the scoring function employed for the ensemble molecular docking. In the first one (Dock1), the default scoring function of QVina2 was used to rank ligand-M^pro^ complexes, while, in the second (Dock2), this value was divided by ${Num\_NoH}^{0.3}$, where Num_NoH represents the number of non-hydrogen atoms of the ligand [34].

(1) LE =affinity/Num_NoH

(2) $$SILE=affinity/{Num\_NoH}^{0.3}$$

As documented in previous studies [55,56,57], ligand efficiencies (LE, Equation (1)) are strongly dependent on system size. Thus, as some authors propose [34,55], the introduction of a correction term in ligand efficiency measures seems to be a good strategy for obtaining size-independent affinity results, not only in hit-to-lead experimental optimization studies, but also in in silico free binding energy estimations.

Ligand efficiency tends to notably decrease with ligand size. Thus, the introduction of size-independent ligand efficiency (SILE, Equation (2)) can result in better drug design experimental processes and promising protein–ligand molecular docking applications [34].

#### 4.1.3. Binding Free Energy Calculations

Binding free energies were computed following the MMPBSA and the MMGBSA approaches [35], as implemented in the AMBER18 package [42]. For both methodologies, the free binding energy is derived from Equation (3), where ${∆H}^{\mathrm{gas}}$ is the gas–phase interaction energy that includes the noncovalent van der Waals (${∆H}_{vdW}^{gas}$) and electrostatic (${∆H}_{elec}^{gas}$) molecular mechanics energies, and the contribution of the polar (${∆G}_{polar}^{solv}$) and non-polar (${∆G}_{nonpolar}^{solv}$) terms corresponds to the solvation free energy (${∆G}^{\mathrm{solv}}$).

(3) $${∆G}_{\mathrm{binding}}={∆H}^{\mathrm{gas}}+{∆G}^{\mathrm{solv}}-T{∆S}^{gas}$$

Therefore, ${∆G}_{polar}^{solv}$ can be numerically calculated with the Poisson–Boltzmann (PB) equation [58], or using the Generalized Born (GB) method [59], for MMPBSA and MMGBSA algorithms, respectively. More concretely, for the MMGBSA calculation, we used the Onufriev–Bashford–Case (OBC) Generalized Born method (igb = 2) [60]. On the other hand, ${∆G}_{nonpolar}^{solv}$ can be obtained as follows:

(4) $${∆G}_{\mathrm{nonpolar}}^{\mathrm{solv}}=\gamma \mathrm{SASA}+\beta$$

where the Solvent-Accessible Surface Area (SASA) is computed with the LCPO method [61] and the constant values for γ and β were set to 0.00542 kcal/mol·Å^2^ and 0.92 kcal/mol for MMPBSA [53] and 0.0072 kcal/mol·Å^2^ and 0 kcal/mol for MMGBSA [54]. In the present project, all binding free energy calculations were performed with the python program MMPBSA.py [62].

The contribution of each M^pro^ protein residue to the total binding free energy of the active compound was analyzed using the MMGBSA decomposition protocol [63] implemented in the MMPBSA.py module of AmberTools20 [64]. The binding interaction for each residue–residue pair includes three contributing terms: Van der Waals, electrostatic, and solvation contribution. The polar contribution of ${∆G}^{\mathrm{solv}}$ was computed as in the case of the ${∆G}_{\mathrm{binding}}$, using the generalized Born model based on the parameters developed by Onufriev et al. [60]. All energy components were calculated using 2500 snapshots corresponding to the last 100 ns of the full-length molecular dynamics run.

#### 4.1.4. Conventional Molecular Dynamics

After minimization, each of the selected complexes were heated to 300 K at a constant rate of 15 K every 10 ps under the canonical ensemble (NVT, heating). A harmonic positional restriction of 1.0 kcal/mol·Å^−2^ for the main atoms of the protein, using the Langevin thermostat algorithm with a collision frequency of 3 ps^−1^, was employed for the purpose.

Once the system was heated, 500 ps of simulation were performed at constant pressure (NPT ensemble), while keeping the main atoms of the protein fixed with a harmonic positional restriction of 1.0 kcal/mol·Å^−2^ for density equilibration. Subsequently, cMDs of different lengths were iteratively carried out under the canonical ensemble to study the complex stability. All simulations were carried out using AMBER18 software package (2018 version) [42].

#### 4.1.5. Gaussian Accelerated Molecular Dynamics

Alternatively, Gaussian accelerated molecular dynamics (GaMD) is a recently developed approach that allows for unconstrained enhanced sampling that does not require the introduction of predefined collective variables [65]. To do so, a boost potential constructed employing a harmonic function that follows a Gaussian distribution is applied to smoothen the Potential Energy Surface (PES) of the system. This smoothing is then introduced only when the potential energy of the system $V\left(\vec{r}\right)$ is lower than a reference energy E_ref_, as depicted in Equations (5) and (6), where $∆V^{*}\left(\vec{r}\right)$ corresponds to the modified system potential.

(5) $$∆V^{*}\left(\vec{r}\right)=V\left(\vec{r}\right)+∆V(\vec{r})$$

(6) $$∆V\left(\vec{r}\right)=\left\{\begin{array}{ll} \frac{1}{2}{k\left(E-V\left(\vec{r}\right)\right)}^{2} & V\left(\vec{r}\right)<E_{ref}\\ 0 & V\left(\vec{r}\right)\geq E_{ref} \end{array}\right.$$

Furthermore, it is important to note that the boost potential added can have two main contributions, implying the modification of the dihedral and total potential energetic terms. This boost potential is computed based on statistics of the system potential, including the minimum, maximum, average, and standard deviation. For the present study, a dual boost was employed for all GaMD simulations.

In this work, GaMD simulations of increasing length were carried out within the NVT ensemble after the initial cMD simulations. In these simulations, an intermediate step was conducted to obtain the initial statistical analysis of the dual boost potential to be applied. The length of this step will vary depending on the number of atoms of the system. For the target evaluated in this study, the upper limit of the standard deviation of the total potential boost (σ0P) was set to 6 and the upper limit of the standard deviation of the dihedral potential boost (σ0V) was set to 6. In all these simulations, a cutoff of 10 Å was employed.

### 4.2. Experimental Procedure

#### 4.2.1. SARS-CoV-2 M^pro^ Expression

M^pro^ was expressed in transformed BL21 (DE3) Gold *E. coli*. After small-scale growth in LB/ampicillin (100 μg/mL) at 37 °C overnight, 4 L LB/ampicillin medium (100 μg/mL) was inoculated and incubated at 37 °C until reaching the induction step (OD at 600 nm around 0.6). Induction was initiated by adding 1 mM isopropyl 1-thio-β-D-galactopyranoside (IPTG) and the culture was incubated at 18 °C for 5 h. Cells were sedimented by centrifugation at 4 °C for 10 min at 10,000 rpm (Beckman Coulter Avanti J-26 XP Centrifuge, Barcelona, Spain) and resuspended in lysis buffer (sodium phosphate 50 mM, pH 7, sodium chloride 500 mM). Cells were lysed by sonication (Sonics Vibra-Cell Ultrasonic Liquid Processor, Newtown, CT, USA) on ice, adding benzonase 20 U/mL (Merck-Millipore, Madrid, Spain) and lysozyme 0.5 mg/mL (Carbosynth, Compton, UK). Centrifugation at 4 °C for 30 min at 20,000 rpm and filtration (0.45 μm-pore membrane) were performed to remove cell debris and polish sample before chromatography. Protein was purified by immobilized metal affinity chromatography (ÄKTA FPLC System, GE Healthcare Life Sciences, Barcelona, Spain) using a cobalt HiTrap TALON column (GE-Healthcare Life Sciences) in a single step, applying an imidazole 10–250 mM gradient. Purity was assessed by SDS-PAGE. Protein fractions were pooled and dialyzed in final buffer (sodium phosphate 50 mM, pH 7, sodium chloride 150 mM). In order to determine the concentration of protein, an extinction coefficient of 32,890 M^−1^ cm^−1^ at 280 nm was employed.

#### 4.2.2. SARS-CoV-2 M^pro^ Proteolytic Activity Assay

The activity of M^pro^ was measured in vitro using the substrate (Dabcyl)KTSAVLQSGFRKME(Edans)-NH2 (Biosyntan GmbH, Berlin, Germany), which contains two fluorophores capable of interacting through Förster resonance energy transfer (FRET). After initiating the reaction by adding substrate at 20 μM (final concentration) to the enzyme at 0.2 μM (final concentration) for a final volume of 100 μL in buffer sodium phosphate 50 mM, pH 7, and NaCl 150 mM, the catalytic activity was recorded through a continuous assay. As compounds were dissolved at high concentration in 100% DMSO, a constant final DMSO percentage (2.5%) was kept in all assays. The FRET effect (fluorescence emission of donor group, which already increased upon substrate cleavage) was measured using a fluorescence microplate reader (FluoDia T70, Photon Technology International, Birmingham NJ, USA) for 20 min (excitation wavelength, 380 nm: emission wavelength, 500 nm). The enzymatic activity and initial enzymatic rate were measured as the initial slope in the fluorescence emission vs. time plot. The Michaelis–Menten constant, *K*_m_, and the catalytic rate constant or turnover number, *k*_cat_, were previously estimated (*K*_m_ = 11 μM, and *k*_cat_ = 0.040 s^–1^) [66].

#### 4.2.3. SARS-CoV-2 M^pro^ Inhibition Assay

The inhibition potency of the compounds against M^pro^ was assessed in vitro using same the enzymatic activity protocol described above, which allowed the estimation of the inhibition constant, *K*_i_, and the half-maximal inhibitory concentration, *IC*50. Inhibition curves were obtained by measuring the enzyme activity (at fixed 0.2 μM enzyme concentration and fixed 20 μM substrate concentration) as a function of compound concentration (serial 2-fold dilution from 125 µM to 0 μM), while maintaining the percentage of DMSO constant (2.5%). The enzymatic activity (initial slope of the fluorescence emission vs. time plot) was measured as a function of compound concentration. The quotient between the activity in the presence/absence of compound provided the residual enzymatic activity percentage at a given compound concentration. Employing a simple inhibition model, the apparent inhibition constant for each compound, *K*_i_^app^, was estimated by non-linear regression analysis according to the following:

(7) $$\left[EI\right]=\frac{1}{2}\left({\left[I\right]}_{T}+{\left[E\right]}_{T}+K_{i}^{app}-\sqrt{{\left({\left[I\right]}_{T}+{\left[E\right]}_{T}+K_{i}^{app}\right)}^{2}-4{{\left[E\right]}_{T}\left[I\right]}_{T}}\right)$$

(8) $$\left[I\right]={\left[I\right]}_{T}-\left[EI\right]=\frac{1}{2}\left({\left[I\right]}_{T}-{\left[E\right]}_{T}-K_{i}^{app}+\sqrt{{\left({\left[I\right]}_{T}+{\left[E\right]}_{T}+K_{i}^{app}\right)}^{2}-4{{\left[E\right]}_{T}\left[I\right]}_{T}}\right)$$

(9) $$\frac{v\left(\left[I\right]\right)}{v\left(\left[I\right]=0\right)}=1-\frac{\left[EI\right]}{{\left[E\right]}_{T}}=\frac{1}{1+\frac{\left[I\right]}{K_{i}^{app}}}$$

where [*EI*] is the concentration of the enzyme-inhibitor complex, [*E*]*_T_* and [*I*]*_T_* are the total concentrations of enzyme and inhibitor, and *v* is the initial slope of the enzymatic activity trace at a given (free) inhibitor concentration [*I*]. No approximations were considered in this model, thus having general validity for any total enzyme and inhibitor concentration and any value of the inhibition constant. If the inhibitor acts competitively with regard to the substrate

(10) $$\frac{v\left(\left[I\right]\right)}{v\left(\left[I\right]=0\right)}=\frac{1}{1+\frac{\left[I\right]}{K_{i}^{app}}}=\frac{1}{1+\frac{\left[I\right]}{K_{i}\left(1+\frac{\left[S\right]}{K_{m}}\right)}}$$

where *K*_i_ is the intrinsic (i.e., substrate concentration-independent) inhibition constant, and [*S*] is the substrate concentration. Neglecting compound depletion (i.e., approximating the free compound concentration by the total compound concentration), the *K*_i_^app^ in Equation (2) is equivalent to the *IC*50. Contrary to *K*_i_^app^, *IC*50 is assay-dependent (depending on [*E*]_T_, [*S*], and *K*_m_) and must be employed cautiously when comparing inhibition potencies.

## 5. Conclusions

The present work, aimed at the identification of inhibitors of the SARS-CoV-2 M^pro^ main protease, proposed an alternative virtual screening docking protocol based on the introduction of a size-independency correction to the original QVina2 scoring function. To do so, the Dark Chemical Matter (DCM) database of drug-like compounds [29] was employed on two independent ensemble docking strategies. As a result, from both independent approaches, a total of seven compounds were selected for in vitro testing. Only four of them were finally subjected to experimental assays, of which one was found to be active.

In the end, our identification of one experimentally active compound, out of four compounds tested, is proof that (1) DCM compounds are worth exploring as a source of new compounds with biomedical interest and (2) our main methodology suffices to identify compounds that are potentially active against selected targets [18,20,21]. Furthermore, based on the good energetic results of compounds that were selected but not experimentally tested, our success rate (one out of four tested compounds, 25%) may be an underestimate. As such, our results warrant the use of our approach in much larger drug discovery efforts.

## Data Availability

Data are contained within the article and Supplementary Materials.

## Supplementary Materials

The following supporting information can be downloaded at https://www.mdpi.com/article/10.3390/ijms25116119/s1.

## Abbreviations

Throughout the present work, certain abbreviations and symbols have been used frequently. Consequently, this table provides a comprehensive list of all the abbreviations utilized throughout the text, accompanied by their respective definition, listed in alphabetical order.

**Abbreviation**	**Definition**	**Page ***
BL21(DE3) Gold *E. Coli*	BL21 (DE3) Chemically Competent Escherichia Coli Cells Derivative	11
ChEMBL	European Molecular Biology Laboratory	2
cMD	Conventional Molecular Dynamics	4
COVID-19	Coronavirus Disease 2019 (2019 novel coronavirus)	1
DCM	Dark Chemical Matter	1
DMSO	Dimethyl Sulfoxide	11
FDA	Food and Drug Administration	2
FRET	Förster Resonance Energy Transfer	11
GaMD	Gaussian Accelerated Molecular Dynamics	4
GAFF2	General Amber Force Field 2	8
GB	Generalized Born	3
*IC*50	Half-Maximal Inhibitory Concentration	12
IPTG	Isopropyl 1-thio-β-D-galactopyranoside	11
*k*_cat_	Catalytic rate constant	12
*K_i_*	Substrate Concentration-Independent Inhibition Constant	1
*K*_i_^app^	Apparent Substrate Concentration-Independent Inhibition Constant	12
*K*_m_	Michaelis–Menten Constant	12
LCPO	Linear Combination of Pairwise Overlaps	10
LB/ampicillin	Luria Broth/Ampicillin Bacterial Culture Medium	11
LE	Ligand Efficiency	9
MMPBSA	Molecular Mechanics Poisson–Boltzmann Surface Area	3
MMGBSA	Molecular mechanics Generalized Born Surface Area	3
M^pro^	Main Protease	1
NVT ensemble	Canonical Ensemble: constant particle number (N), constant volume (V) and temperature fluctuating around an equilibrium value (T).	8
OBC	Onufriev–Bashford–Case	10
OD	Optical Density	11
OPC	Four-Point Optimal Point Charge Water Model	8
PB	Poisson–Boltzmann	3
PDB	Protein Data Bank	8
PES	Potential Energy Surface	10
RNA	Ribonucleic Acid	2
SASA	Solvent-Accessible Surface Area	10
SARS-CoV-2	Severe Acute Respiratory Syndrome Coronavirus 2	1
SDS-PAGE	Sodium Dodecyl Sulfate–Polyacrylamide Gel Electrophoresis	11
SILE	Size-Independent Ligand Efficiency	9
TIP3P	Transferable Intermolecular Potential with 3 Points	8
VS	Virtual Screening	8
ZINC	ZINC is not commercial	2
* Page number corresponds to the first appearance of the abbreviation in the text.		
