# Supplementary material for: Shedding Light on Dark Chemical Matter: The Discovery of a SARS-CoV-2 Mpro Main Protease Inhibitor through Intensive Virtual Screening and In Vitro Evaluation
Source: Int J Mol Sci. 2024 Jun 1;25(11):6119. doi: 10.3390/ijms25116119 (PMC11172690; doi:10.3390/ijms25116119)
Supplement: Supplementary file 1 [file ijms-25-06119-s001.zip › ijms-3016398-supplementary.pdf]

Supporting information

Shedding Light on Dark Chemical Matter: The Discovery of a SARS-CoV-2 Mpro Main Protease Inhibitor through Intensive Virtual Screening and In Vitro Evaluation

Maria Nuria Peralta-Moreno [1], Yago Mena [1], David Ortega-Alarcon [2,3], Ana Jimenez-Alesanco [2,3], Sonia Vega [2], Olga Abian [2,3,4,5], Adrian Velazquez-Campoy [2,3,4,5], Timothy M. Thomson [5,6,7], Marta Pinto [8], José M. Granadino-Roldán [9], Maria Santos Tomas [10], Juan J. Perez [11] and Jaime Rubio-Martinez [1,*]

[1] Department of Materials Science and Physical Chemistry, University of Barcelona (UB), and the Institut de Recerca en Quimica Teòrica i Computacional (IQTCUB), 08028 Barcelona, Spain.

[2] Institute of Biocomputation and Physics of Complex Systems (BIFI), Joint Unit GBsC-CSIC-BIFI, Universidad de Zaragoza, 50018 Zaragoza, Spain.

[3] Departamento de Bioquímica y Biología Molecular y Celular, Universidad de Zaragoza, 50009 Zaragoza, Spain.

[4] Instituto de Investigación Sanitaria de Aragón (IIS Aragon), 50009 Zaragoza, Spain.

[5] Centro de Investigación Biomédica en Red en el Área Temática de Enfermedades Hepáticas Digestivas (CIBERehd), 28029 Madrid, Spain.

[6] Institute of Molecular Biology of Barcelona (IBMB-CSIC), 08028 Barcelona, Spain.

[7] Instituto de investigaciones de la Altura, Universidad Peruana Cayetano Heredia, Av. Honorio Delgado 430, Lima 15102, Perú.

[8] AbbVie Deutschland GmbH & Co. KG, Computational Drug Discovery, Knollstrasse, 67061 Ludwigshafen, Germany.

[9] Departamento de Química Física y Analítica, Facultad de Ciencias Experimentales, Universidad de Jaén, Campus "Las Lagunillas" s/n, 23071 Jaén, Spain.

[10] Department of Architecture Technology, Universitat Politecnica de Catalunya (UPC), Av. Diagonal 649, 08028 Barcelona, Spain.

[11] Department of Chemical Engineering, Universitat Politecnica de Catalunya (UPC) - Barcelona Tech. Av. Diagonal, 647, 08028 Barcelona, Spain.

* Corresponding author's e-mail: jaime.rubio@ub.edu

Table S1. Maximum and minimum scoring function values obtained for each of the 7 selected SARS-CoV-2 M^{pro} representative structures, performed with the two independent ensemble docking processes (Dock1 and Dock2). Units in kcal/mol.

		cMD_c0	cMD_c1	cMD_c2	GaMD_c0	GaMD_c1	GaMD_c2	GaMD_c3
Dock1	Max	-8.7	-9.5	-9.2	-9.0	-9.6	-9.1	-9.0
	Min	-3.0	-2.4	-2.5	-2.8	-2.7	-2.9	-2.7
Dock2	Max	-3.0	-3.2	-3.2	-3.0	-3.3	-3.1	-3.1
	Min	-1.1	-0.8	-0.8	-0.9	-0.9	-1.0	-0.9

Table S2. Thresholds stablished for the docking scoring function and number of generated poses selected for each of the 7 selected SARS-CoV-2 M^{pro} representative structures, performed with the two independent ensemble docking processes (Dock1 and Dock2). Units in kcal/mol.

		cMD_c0	cMD_c1	cMD_c2	GaMD_c0	GaMD_c1	GaMD_c2	GaMD_c3
Dock1	Energy threshold	-8.1	-8.1	-8.1	-8.1	-8.1	-8.1	-8.1
	Selected structures	95	630	212	88	2097	387	116
Dock2	Energy threshold	-2.9	-3.1	-3.0	-2.9	-3.2	-3.0	-3.1
	Selected structures	202	178	99	147	112	151	123

Table S3. Number of compounds selected for structure minimization obtained from the two different docking processes (Dock1 and Dock2) for each of the selected SARS-CoV-2 M^{pro} representative structures.

	cMD_c0	cMD_c1	cMD_c2	GaMD_c0	GaMD_c1	GaMD_c2	GaMD_c3
Dock1	62	432	126	63	1121	239	83
Dock2	177	162	87	120	98	127	109

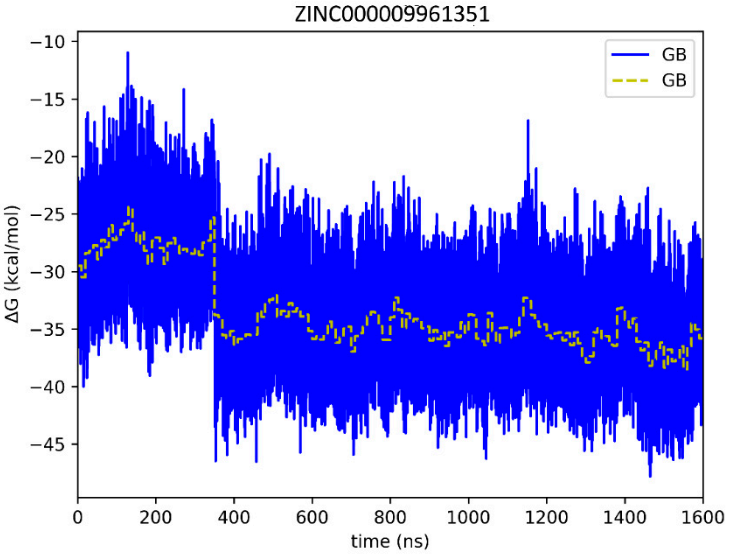

Figure S1. Time evolution of the binding free energy of the active compound selected from the virtual screening process.

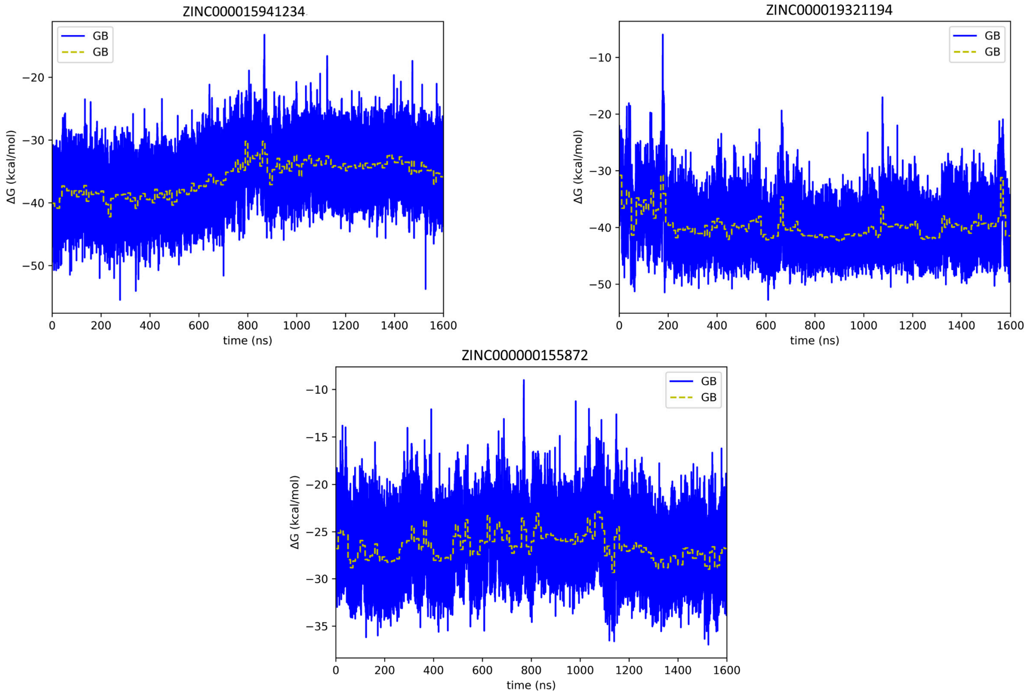

Figure S2. Time evolution of the binding free energy of the tested but non-active compounds selected from the virtual screening process.

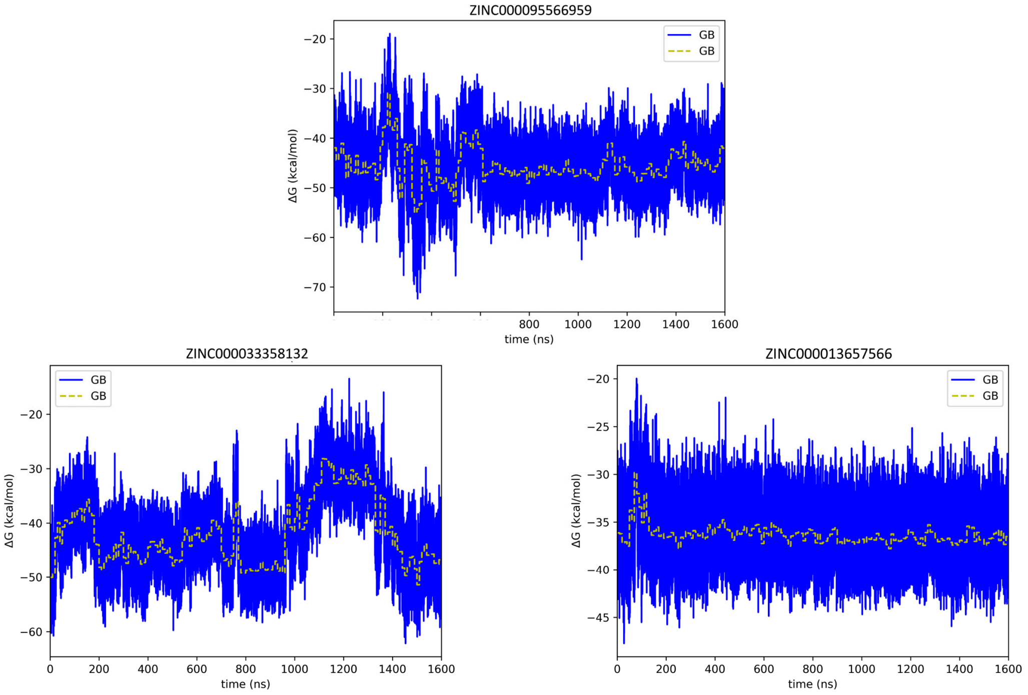

Figure S3. Time evolution of the binding free energy of the non-available compounds selected from the virtual screening process.

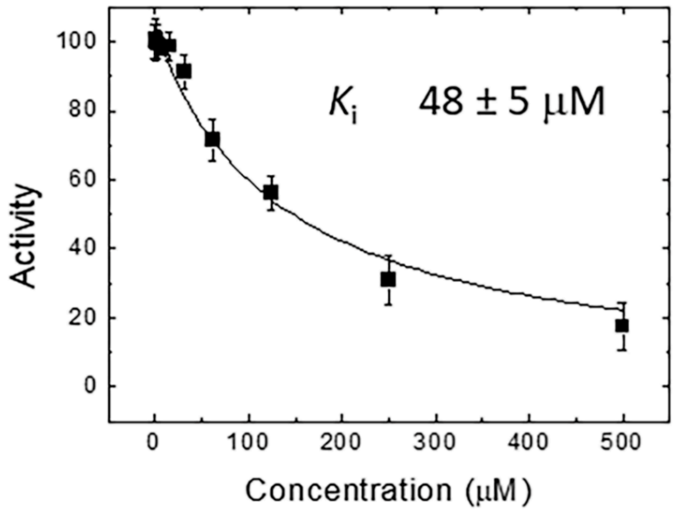

Figure S4. *In vitro* M^pro^ inhibitory activity of the active compound. Serial dilutions of the compound were used in a FRET-based activity assay with recombinant SARS-CoV-2 M^pro^ and a two-fluorophore substrate amenable to FRET. The (substrate concentration-independent) inhibition constant, K_i, was calculated as described in *Materials and Methods* section.

Table S4. Average binding free energy during the last 50 ns and 100ns of extended Molecular Dynamics simulations for all the selected compounds. The experimentally tested compounds are highlighted in grey. In bold, the experimentally active compound. Units in kcal/mol. DM refers to Dark Matter.

Compound	ZINC ID	$\Delta G_{binding}$ (GB)	
		50 ns	100 ns
**DM1**	**ZINC000009961351**	**-36.1**	**-36.7**
DM2	ZINC000015941234	-35.6	-35.1
DM3	ZINC000095566959	-44.3	-44.8
DM4	ZINC000033358132	-46.6	-46.7
DM5	ZINC000019321194	-38.1	-38.8
DM6	ZINC000013657566	-36.5	-36.7
DM7	ZINC000000155872	-27.6	-27.7

Table S5. Major binding free energy contributions obtained from the last 100 ns of 1.6 µs GaMD trajectory for the active compound DM1. Only those protein residues with a value greater than -1.0 kcal/mol are reported. σ: Standard deviation. $\bar{\sigma}$: Mean Standard Deviation. Units in kcal/mol.

Residue	ΔG (kcal/mol)	σ	$\bar{\sigma}$
H41	-5.320	1.129	0.023
M49	-1.192	1.170	0.023
H164	-1.785	0.320	0.006
M165	-4.829	0.676	0.014
P168	-1.087	0.383	0.008
D187	-3.576	0.357	0.007
R188	-1.184	0.203	0.004
Q189	-8.167	0.720	0.014
T190	-7.440	0.774	0.015
A191	-1.535	0.231	0.005
Q192	-2.038	0.314	0.006

Table S6. Description of the most relevant hydrogen bonds obtained from the last 100 ns of the 1.6 µs GaMD trajectory for the active compound in complex with the SARS-CoV-2 M^{pro} main protease.

System DM1	Acceptor	Donor	Occupancy (%)
ZINC000009961351<br>CAS 877126-22-2	Ligand_Oc	THR190_N-H	88.4
	GLN189_OE1	Ligand_Nb-Hb	78.8
	THR190_O	Ligand_Na-Ha	55.4

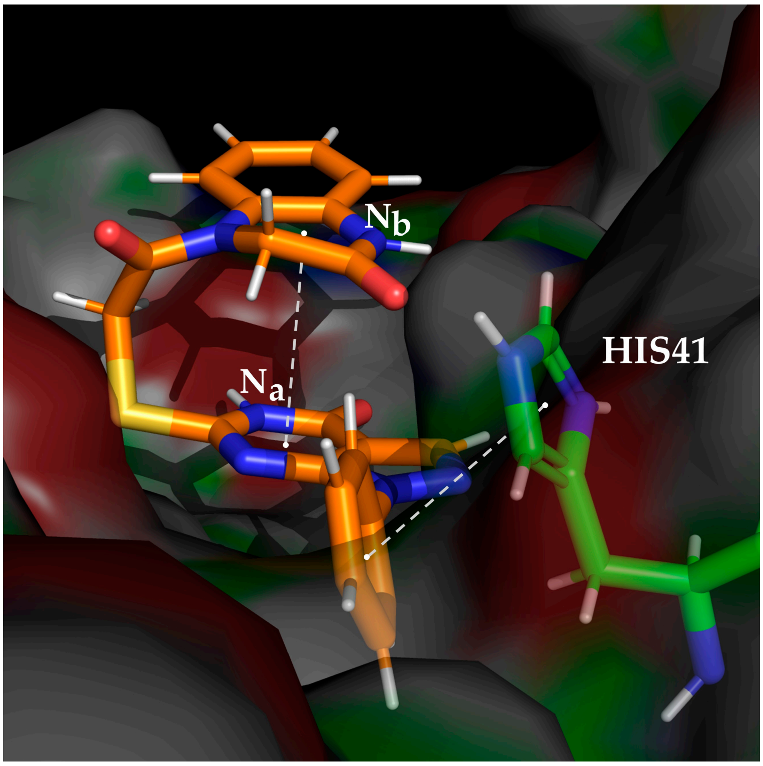

Figure S5. Three-dimensional representation of compound DM1 (orange) in complex with the SARS-CoV-2 M^{pro} main protease, obtained from the last snapshot of the extended 1.6 μs GaMD simulations. Depicted in white dashed lines, intramolecular π-π interactions help the ligand to adopt its bioactive conformation. Also, π-π interactions between H41 (green) and benzene group of DM1 can be observed.
